# The prevalence of peripheral neuropathy among the patients with diabetes in Pakistan: a systematic review and meta-analysis

**DOI:** 10.1038/s41598-023-39037-1

**Published:** 2023-07-20

**Authors:** Sohail Akhtar, Fazal Hassan, Seda Rakhshanda Saqlain, Aqsa Ali, Sardar Hussain

**Affiliations:** 1 Department of Mathematics and Statistics, The University of Haripur, Haripur, KP Pakistan; 2 Department of Statistics, GC University Lahore, Lahore, Punjab Pakistan; 3 Department of Statistics, Quaid Azam University, Islamabad, Pakistan

**Keywords:** Endocrinology, Health care

## Abstract

The most frequent complication of diabetes is peripheral neuropathy. The estimated prevalence of peripheral neuropathy in people with diabetes varies substantially between published studies in Pakistan. We conducted this meta-analysis to summarize the prevalence of peripheral neuropathy in people with diabetes. Different electronic databases were systematically searched using keywords and MeSH terms. Random-effects meta-analysis was conducted to pool the prevalence of peripheral neuropathy in people with diabetes in Pakistan. Heterogeneity was investigated by random-effects meta-regression and stratification. Two independent authors reviewed studies, extracted data, and conducted the risk of bias analysis. Nineteen studies with a total of 8487 diabetic patients were included. The overall pooled prevalence of diabetic peripheral neuropathy was 43.16% (95% CI 32.93–53.69%), with significant heterogeneity between estimates. The prevalence of peripheral neuropathy among those newly diagnosed with diabetes was 26.52% (95% CI 14.97–39.96%, n = 5). According to the subgroup meta-analysis, the pooled prevalence of diabetic peripheral neuropathy was highest in Khyber Pakhtunkhwa (55.29%; 95% CI 23.91–84.50%), followed by Sindh (40.04%; 95% CI 24.00–57.25%), and the lowest was found in Punjab (34.90%; 95% CI 15.05–57.95%). A significant association was found between the pooled prevalence estimate and the duration of diabetes. The results of this meta-analysis indicate a relatively high prevalence of peripheral neuropathy in people with diabetes in Pakistan. The study protocol has been registered in the PROSPERO, with the registration number CRD42022371617.

## Introduction

Diabetes mellitus is a serious disease that persists over time and has an enormous negative influence on people's lives, families, and communities of people all over the world^1^. The International Diabetes Federation (IDF) 2021 reported that there are currently 537 million people with diabetes and predicts this number will increase to 643 million in 2030 and 783 million by 2045^2^. Diabetes was responsible for an estimated 1.6 million deaths in 2015^2^. Over the few decades, more significant increases in prevalence have been observed in countries with lower and middle incomes than in nations with higher incomes^2^. Furthermore, it was estimated that around 6.7 million individuals aged 20 to 79 will die because of diabetic complications in 2021^3^. Diabetes increases the risk of complications from a variety of medical illnesses. Consistently high blood sugar levels cause cardiovascular and blood vessel disease, eye and kidney illness, nerve damage (neuropathy), and tooth decay^4^. Diabetes can cause nerve damage if blood sugar and blood pressure levels are excessively high^5^. This can cause issues with digestion, erectile dysfunction, and a variety of other processes^5^. Among the most frequently affected regions are the extremities, specifically the feet^6^. Diabetic peripheral neuropathy is damage to the nerves in the extremities, usually the feet and legs, due to diabetes^7^. Symptoms can include numbness, tingling, pain, and weakness. Peripheral neuropathy resulting from diabetes significantly contributes to disability worldwide^8^, reducing the quality of life due to sensory loss, an increased risk of falling^9^, an increased risk of foot ulcerations^10^, limb amputation^11^, and increased treatment expenses^12^. Furthermore, diabetic peripheral neuropathy symptoms are frequently associated with sleep problems, anxiety, and sadness^13^. In the patients with diabetes, peripheral nerve degeneration is typically irreversible^14^. This has prompted healthcare practitioners to prioritize preventing and identifying modifiable risk factors^14^.

The increasing number of cases of diabetes and the problems it causes are becoming a primary concern in Pakistan, affecting the health and well-being of individuals and families^15–20^. According to the IDF 2021, Pakistan has the highest prevalence (26.3%) of diabetes globally. It has on 3rd highest number of the patients with diabetes after China (140 million) and India (74 million), with 1 out of 4 adults living with diabetes in the country^21^. Diabetes is the major cause of peripheral neuropathy in Pakistan. Published studies have reported the prevalence of peripheral neuropathy in people with diabetes in Pakistan varies substantially that, ranging from 16.30%^22^ to 79.50%^23^. Thus, this study aimed to systematically gather and summarize existing data on the prevalence of peripheral neuropathy among patients with diabetes in Pakistan. To the best of our knowledge, no prior research has attempted to pool the data on the prevalence of diabetic peripheral neuropathy in Pakistan.

## Methods

This study adhered to the Preferred Reporting Items for Systematic Reviews and Meta-analyses (PRISMA) guidelines^24^. The protocol for this review has been registered in the PROSPERO International Prospective Registry of Systematic Reviews with the registration number CRD42022371617 (Supplementary Information).

### Search strategy

We extensively searched for publications on the prevalence of diabetic peripheral neuropathy in Pakistan, using multiple databases, including Medline via PubMed, Web of Science, Google Scholar, and local Pakistani databases. The search included publications from inception till December 30th, 2022. We combined medical subject terms with search phrases such as “diabetes and peripheral neuropathy”, “diabetic peripheral neuropathy”, “diabetic neuropathy”, “DPN”, diabetic complication”, “prevalence” and “Pakistan”. The last search was conducted on 31 December 2022. Two authors (S.A. and F.H.) independently screened all publications for eligibility, with differences addressed by a third author (S.R.S.).

### Inclusion and exclusion criteria

Studies were considered for inclusion in the quantitative synthesis if they fulfilled the criteria outlined: (i) reported the prevalence or provided enough information to calculate it for diabetic peripheral neuropathy; (ii) were based on a hospital, population, or community surveys; and (iii) were written in English. Studies were excluded if they: (i) were not related to peripheral neuropathy in people with diabetes; (ii) were reviews; (iii) were case series or case reports; (iv) were about Pakistani communities outside of Pakistan; (v) had data that was published in more than one study (the most recent version was used); and (vi) did not have full text available.

### Risk of bias assessment

Using the Joanna Briggs Institute Critical Appraisal^25^ checklist, two authors (F.H. and A.A.) independently assessed the methodological quality of each selected study. Disputes over the quality evaluation checklist were handled by discussion or consulting a third investigator (S.R.S.). Each study was rated as good (scores greater than 69%), medium (scores between 50 and 69%), or poor (scores below 50%).

### Data extraction

Two independent authors (A.A. and F.H.) obtained the following information from each included study using a standardized form: The following data is abstracted: Surname of the first author, year of publication, prevalence, study design, sample size, setting, geographic location, type of diabetes, sex (male or female), the percentage of male patients, the working year, the mean age of the patients, sampling method, risk of bias, the percentage of diabetes and hypertensive patients, the percentage of the patients who were smokers, the percentage of patients who were obese or overweight, and the prevalence of diabetes in the participants' families. A third author (S.A.) resolved disagreements by discussion and adjudication.

### Statistical analysis

Random-effects (DerSimonian and Laird) models were employed because of the assumed heterogeneity between the studies. The statistical analysis was performed through the statistical software R and two packages (meta and metafor). Before combining prevalence estimates, Freeman-Tukey double arcsine transformation^26,27^ of percentage was applied to stabilize variances. Furthermore, for all pooled estimates, 95% prediction intervals were computed^27^. A Forest plot was produced to visualize pooled point estimates and their respective confidence interval (CIs). Between-study heterogeneity was quantified through the I^2^ index and tested using Cochran *Q* statistics^28^. Publication bias (small study effect) was visually inspected through a funnel plot and quantitatively tested using the Egger^29^ and Begg^30^ correlation tests. We further investigated potential sources of statistical heterogeneity employing meta-regression and subgroup meta-analysis. We did not create a multivariable meta-regression model due to the small number of publications. Two-sided P < 0.10 indicated statistical significance. The amount of total between-study variability explained by covariates in the regression models was assessed using the R^2^ metric. We performed a leave-one-out sensitivity analysis on meta-analysis to check if any study was highly influential^31^. We determined the level of agreement between raters for the study inclusion and the data extraction using Cohen’s kappa coefficient (κ)^32^.

## Results

**Figure 1:**
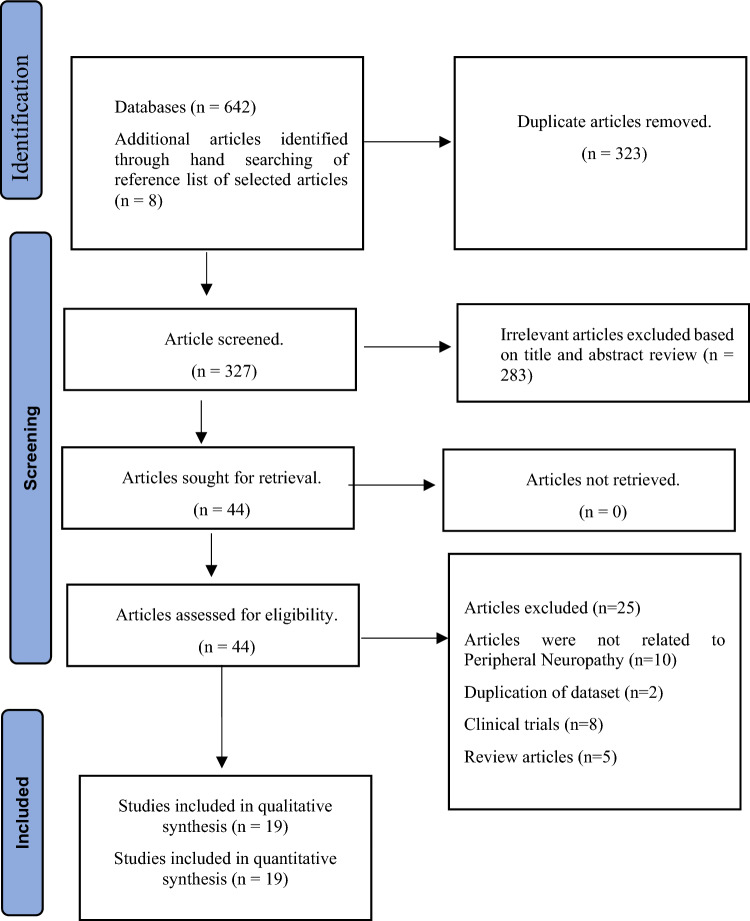
PRIMSA flow chart of the prevalence of DPN in Pakistan^24^.

We identified 642 potential articles from the electronic databases and eight additional articles from the reference list of the identified articles. After screening the titles and abstracts, we deleted 323 duplicate publications and excluded 283 entries, as shown in Fig. 1. There were 44 articles left for full-text review. Finally, we included 19 studies that met the inclusion criteria for this systematic review.

### Study characteristics

Table 1 describes the key characteristics of the included articles in the review. In total, 8487 patients with diabetes were considered in this study. The study's sample sizes ranged from 107^45^ to 1940^29^ patients, with a median of 250 (interquartile range, 150–368) patients. The mean age range of the patients was reported in 18 studies^22,23,34–37,41–49^. The mean age of patients with diabetes in the included articles was between 44.8^48^ and 62.26^40^ years. The proportion of male patients in the study sample was reported in 18 studies^22,23,33–36,41–49^, with a total of 3813 male participants among 8487 participants (a mean of 46.0%). All studies considered a cross-sectional study design. The included articles were published between 2011 and October 2022, while the period of investigations was from June 2014 to February 2021. Three provinces of Pakistan were represented in the included articles: Eight were conducted in Sindh^22,34,39,41,44–46,48^, six in Punjab^35,36,38,43,47,49^, four in Khyber Pakhtunkhwa^23,33,37,40^ and one study at the national level^42^. Furthermore, 17 studies were conducted in urban areas^22,23,33–41,43,45–49^ and one in rural area^44^. The proportion of male patients ranged from 18.07 to 60.8%. The included studies were evaluated methodologically, and 15 were rated moderately biased^22,33–40,42–44,46,48,49^, one was rated low bias^41^, and three were rated high bias^23,45,47^. It was found that there was an inter-rater agreement of 0.81 for study selection and 0.88 for data extraction between the authors.

**Table 1:** General characteristics of the included studies.

Author	Year	Study design	Sample size	Positive cases	Prevalence %	Setting	Province	Diabetes type	Diagnosed	Sex	Male %	Working year	Age	Sampling method	Risk of bias
Aamir et al.^33^	2011	CS	114	70	61.00	Urban	KP	NA	Combined	Both	52	2005–2006	NA	NA	Moderate
Phulpoto et al.^34^	2012	CS	1044	364	34.90	Urban	Sindh	NA	Combined	Both	50.96	2010–2011	53.3	NA	Moderate
Iftikhar et al.^35^	2014	CS	250	187	74.80	Urban	Punjab	NA	Combined	Both	18.07	NA	49.52	NA	Moderate
Dar et al.^36^	2016	CS	200	88	44	Urban	Punjab	Type-2	Combined	Both	60.50	2015	54.47	NA	Moderate
Ahmad et al.^22^	2017	CS	1280	208	16.30	Urban	Sindh	Type-2	Combined	Both	49.80	2014–2016	54.9	NA	Moderate
Kundi et al.^37^	2018	CS	368	154	39.90	Urban	KP	NA	Combined	NA	NA	2017	56.22	Non-probability sampling	Moderate
Younis et al.^38^	2018	CS	1940	510	26.30	Urban	Punjab	Type-2	Combined	Both	37	2016–2017	51.24	NA	Moderate
Rafi et al.^39^	2019	CS	120	62	51.70	Urban	Sindh	Type-2	Combined	Both	55.80	2018	55.3	NA	Moderate
Tahir et al.^40^	2020	CS	270	99	36.70	Urban	KP	Type-2	Combined	Both	60	2017	62.26	Non-probability sampling	Moderate
Gurbakhshani et al.^41^	2020	CS	327	236	72.20	Urban	Sindh	Both	Combined	Both	34.30	2017–2018	57.2	Systemic random sampling	Low
Hussain et al.^23^	2021	CS	365	290	79.50	Urban	KP	Type-2	Combined	Both	41.10	2019	49	NA	High
Arshad et al.^42^	2021	CS	120	85	71	NA	overall	Type-2	Combined	Both	37	2018–2019	60.42	Simple random sampling	Moderate
Kalam et al.^43^	2021	CS	270	70	26.00	Urban	Punjab	Type-2	Combined	Both	43.70	NA	54.88	Non-probability sampling	Moderate
Sahito et al.^44^	2022	CS	1057	607	57.42	Rural	Sindh	Type-2	Combined	Both	60.80	2020–2021	50.3	NA	Moderate
Lakhair et al.^45^	2014	CS	107	18	16.82	Urban	Sindh	Type-2	Newly	Both	53.30	2011–2012	45.19	NA	High
Naqvi et al.^46^	2018	CS	155	57	37.70	Urban	Sindh	Type-2	Newly	Male	58	2017–2018	49.6	NA	Moderate
Akram et al.^47^	2019	CS	150	31	20.67	Urban	Punjab	Type-2	Newly	Both	54.00	2014–2015	46.35	NA	High
Qureshi et al.^48^	2020	CS	150	59	39.30	Urban	Sindh	Type-2	Newly	Both	60.70	2019	44.8	Non-probability sampling	Moderate
Tahir et al.^49^	2021	CS	200	42	21	Urban	Punjab	Type-2	Newly	Both	18	NA	47.05	Non-probability sampling	Moderate

*KP* Khyber Pakhtunkhwa, *NA* not given, *CS* cross-sectional.

### Pooled prevalence

**Figure 2:**
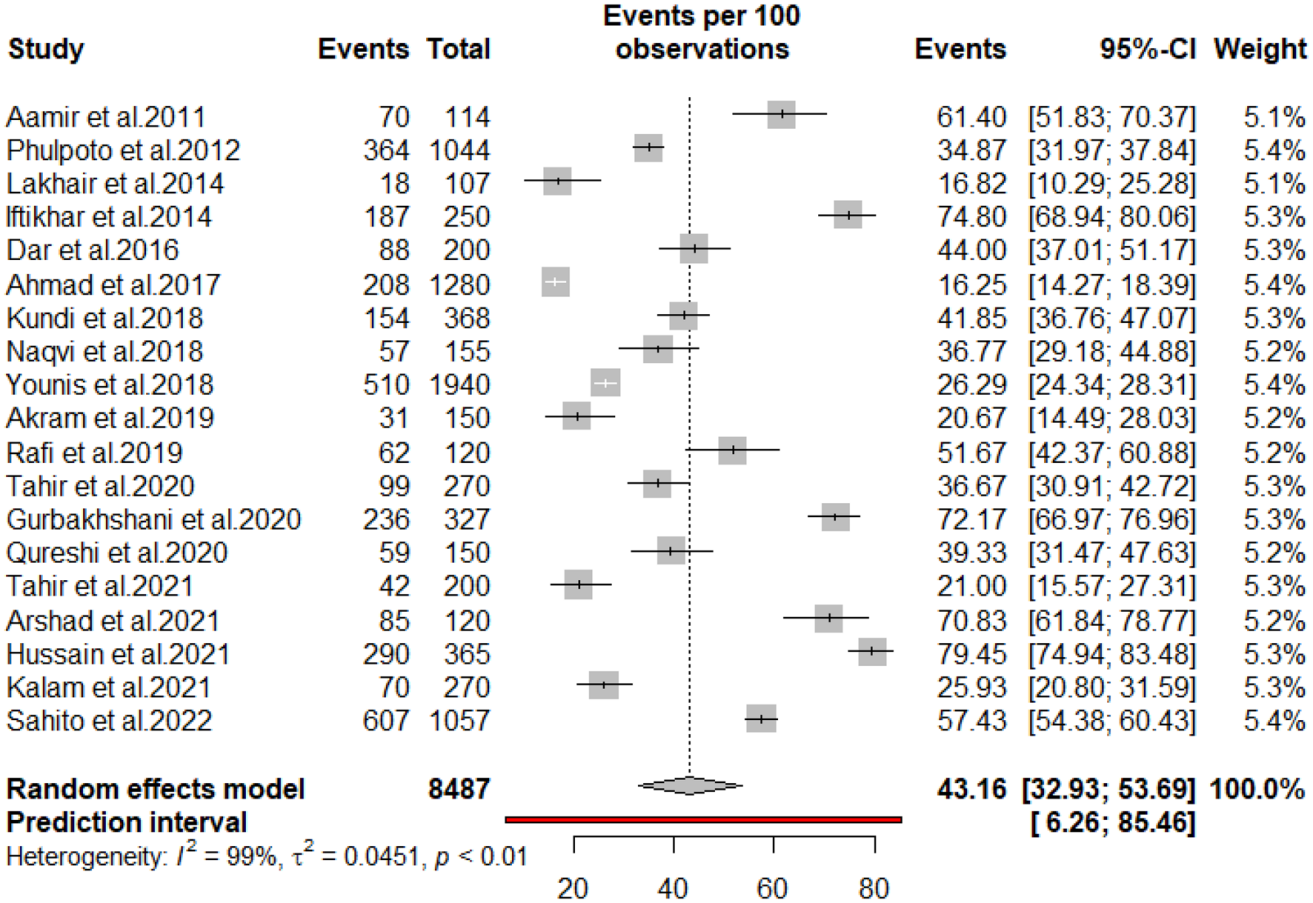
Forest plot of the prevalence of DPN in Pakistan.

**Figure 3:**
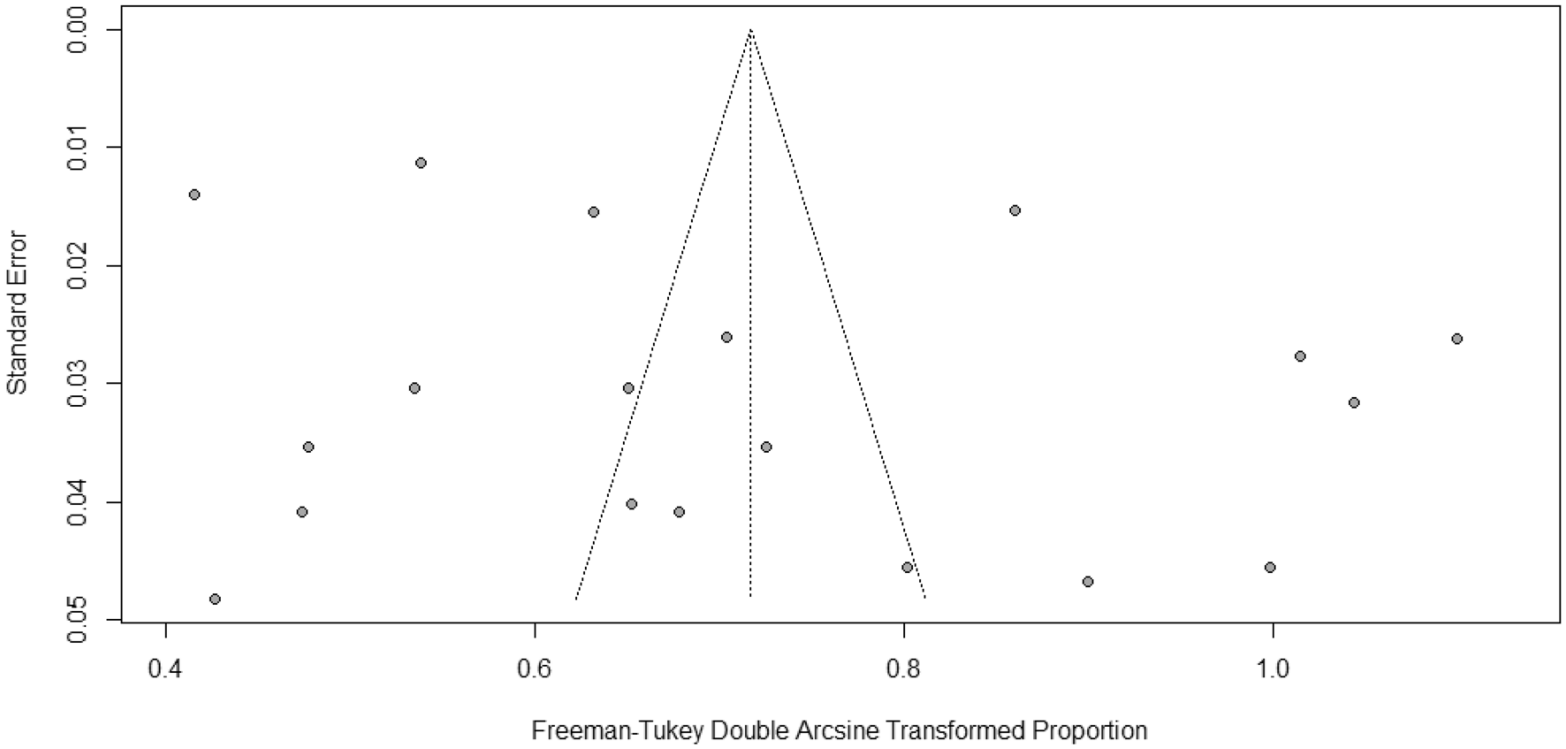
Funnel plot of the prevalence of DPN in Pakistan.

Table 2 presents overall and subgroup meta-analyses of the prevalence of diabetic peripheral neuropathy in Pakistan. Prevalence estimates of diabetic peripheral neuropathy in Pakistan in the included studies varied widely from 16.30% (95% CI 14.27–18.39%) to 79.50% (95% CI 74.94–83.48%). The random-effects overall pooled estimated prevalence of diabetic peripheral neuropathy was 43.16% (95% CI 32.93–53.69%). The 95% prediction intervals were 6.26–85.46% (Fig. 2). High heterogeneity was noted across studies (I^2^ = 98.7%; P < 0.01). The funnel plot (Fig. 3), Begg correlation rank test (z = 0.32, p-value = 0.7523), and Egger’s test (t = 1.18; p = 0.1424) all indicated that the meta-analysis had no publication bias. By removing each study individually, the pooled prevalence of peripheral neuropathy ranged from 41.03% (95% CI 31.98–50.39%) to 44.87% (95% CI 35.28–55.65%). The sensitivity analysis discovered that no single study significantly impacted the pooled prevalence of peripheral neuropathy.

**Table 2:** The prevalence of diabetic peripheral neuropathy in Pakistan, from inception till December 2022.

Variable	Studies	Sample	Cases	Prevalence, % (95% CI)	I^2^, %	95%, Prediction interval	p-Heterogeneity	P-Beggs	p Egger	P-Difference
Peripheral neuropathy	19	8487	3237	43.16 (32.93–53.69)	0.987	(6.26–85.46)	< 0.001	0.7523	0.1424	
Newly diagnosed diabetes	5	762	207	26.52 (14.97–39.96)	0.869	(2.65–62.66)	< 0.001			
By sex								0.0395	0.0178	0.7694
Male	10	1682	865	47.37 (30.47–64.58)	0.966	(5.09–92.28)	< 0.001			
Female	10	1394	792	44.08 (25.00–64.09)	0.966	(1.87–93.21)	< 0.001			
Duration of diabetes								0.6918	0.2672	0.0482
5 years	8	1013	419	34.64 (25.73–54.45)	0.923	(7.03–78.72)	< 0.001			
> 5 years	7	1689	995	56.77 (35.69–76.67)	0.971	(7.88–97.96)	< 0.001			
By location								0.8793	0.2071	0.3033
Punjab	6	3010	928	34.90 (15.05–57.95)	0.978	(0.05–88.38)	< 0.001			
Sindh	8	4240	1611	40.04 (24.00–57.25)	0.990	(0.78–91.48)	< 0.001			
Khyber Pakhtunkhwa	4	1117	613	55.29 (23.91–84.50)	0.982	(0.00–100.0)	< 0.001			
By age								0.3244	0.3765	0.0355
20–40	6	327	88	23.68 (14.02–34.82)	0.738	(0.47–62.80)	0.0015			
41–60	6	994	506	41.90 (27.30–57.24)	0.908	(1.90–90.66)	< 0.001			
61–80	6	590	417	55.50 (28.50–80.94)	0.964	(0.00–100.0)	< 0.001			
Survey period								0.5883	0.1410	0.0367
< 2018	6	2995	935	40.46 (16.85–66.68)	0.987	(0.00–96.70)	< 0.001			
≥ 2018	13	5492	2302	44.42 (32.04–57.15)	0.989	(5.61–88.11)	< 0.001

The prevalence estimates of peripheral neuropathy in newly diagnosed diabetes were observed in 21.0% (5/19) of studies that provided data on peripheral neuropathy ranging from 0 to 80.5%. The pooled prevalence of peripheral neuropathy among newly diagnosed diabetes patients was 26.52% (95% CI 14.97–39.96%). The 95% prediction interval was 2.65–62.66%. The studies had high heterogeneity (I^2^ = 86.9% with P < 0.01).

### Subgroup analysis

Subgroup meta-analysis showed differences in peripheral neuropathy prevalence by the duration of diabetes. Patients with diabetes for more than 5 years were reported to have the highest pooled peripheral neuropathy prevalence estimate (56.77%; 95% CI 35.69–76.67%) than patients having a diabetes history of fewer than 5 years (34.64%; 95% CI 25.73–54.45%). When stratified by geographic locations, the pooled prevalence diabetic peripheral neuropathy estimates were 55.29% (95% CI 23.91–84.50%) in Pakhtunkhwa, 40.04% (95% CI 24.00–57.25%) in Sindh and 34.90% (95% CI 15.05–57.95%) in Punjab.

When stratified by gender, the pooled diabetic peripheral neuropathy in male patients was (47.37%; 95% CI 30.47–64.58%) was slightly higher than in female patients (44.08%; 95% CI 25.00–64.09%), but the difference was statistically insignificant. The prevalence estimates of diabetic peripheral neuropathy in the age groups 20–40, 41–60, and 60–80 were 23.68% (95% CI 14.02–34.82), 41.90% (95% CI 27.30–57.24%) and 55.50% (95% CI 28.50–80.94%), respectively. Diabetic peripheral neuropathy is more prevalent in people over the 60–80 age group, and the prevalence increases with age.

According to univariate meta-regression analysis, only the duration of diabetes of the diabetic patient was statistically significant (β = 0.0211; 95% CI 00.0017–0.0405; P = 0.03, R^2^ = 17.20%). There was no statistically significant relationship between prevalence and publication year, smoking, obesity, hypertension, mean age, sample size, methodological quality, or percentage of males in the sample (Table 3).

**Table 3:** Univariable meta-regression analysis.

Covariate	Co-efficient (*β*)	p-value	95% confidence interval	*R*^2^ (%)
Year of publication	0.0049	0.7635	− 0.0271 to 0.0369	Nil
Age	0.0111	0.2936	− 0.0096 to 0.0318	0.29
Hypertension	− 0.0026	0.6508	− 0.0139 to 0.0087	Nil
Methodology	− 0.0340	0.1234	− 0.080 to 0.033	Nil
Duration of diabetes	0.0211	0.0331	0.0017 to 0.0405	17.20
Smoking	0.0079	0.3830	− 0.0098 to 0.0256	Nil
Sample size	− 0.0001	0.2852	− 0.0003 to 0.0001	0.91
Overweight/obesity	0.0029	0.7013	− 0.0119 to 0.0177	Nil

## Discussion

Diabetes is a chronic condition that makes it difficult for a person to control the amount of sugar in their blood. It is a leading cause of many health complications, including diabetic peripheral neuropathy, a type of nerve damage affecting the feet and legs. As a primary objective of this study, we compiled data on the prevalence of diabetic peripheral neuropathy among and their correlated risk factors in Pakistan. It is anticipated that this meta-analysis will help fight against peripheral neuropathy and its complications by supplying information that will assist in public health efforts. The total number of patients with diabetes in the selected studies was 8487. The overall pooled prevalence of peripheral neuropathy in patients with diabetes was 43.16% (95% CI 32.93–53.69%). This finding is in line with a meta-analysis performed in Latin America and the Caribbean (46.5%)^50^, Saudi Arabia (40.2)^51^, and Africa 46%^52^. Our study found a higher prevalence than similar studies conducted in other countries, which found a prevalence of 35.78%^53^ worldwide (31%)^54^ Ethiopia is 22%^55^. However, the prevalence was lower than that of a similar study performed in Iran, which found a prevalence rate of 53%^56^. It's possible that the discrepancy is due to different diagnostic criteria for diabetic peripheral neuropathy, getting diagnosed early, and starting therapy right away.

In newly diagnosed diabetes patients, the prevalence of peripheral neuropathy was substantially lower than in overall patients. The prevalence rate of peripheral neuropathy was 26.52% among newly diagnosed patients with diabetes. Our results are in line with a recent study (26.1%) conducted on newly diagnosed type-2 diabetes^57^.

Subgroup meta-analysis by study location revealed that the pooled prevalence of peripheral neuropathy in the patients with diabetes was highest in Khyber Pakhtunkhwa (55.29%), followed by Sindh (40.04%) and lowest in Punjab (34.90%). This disparity in the prevalence may be due to socioeconomic and sociocultural differences between populations, variations in screening methods, regional differences, seasonal and climate factors, lifestyle habits, and eating habits. Subgroup meta-analysis by age group revealed that the prevalence of diabetic peripheral neuropathy was significantly higher among the older age patients than among the younger age patients. Diabetic patients aged 60 years and over are twice more likely to have peripheral neuropathy problems than those aged 20–40 years. The findings also showed that the duration of diabetes is one of the significant risk factors for peripheral neuropathy disease. The pooled prevalence of peripheral neuropathy in patients with diabetes with a time duration of more than 5 years (56.77%) is greater than in the patients with less than 5 years of diabetes duration (34.64%). Our results are consistent with earlier studies^58,59^.

Our meta-analysis also showed several limitations. First, we discovered significant heterogeneity between studies, therefore we performed meta-regression and subgroup analysis to identify the sources of heterogeneity and an adjusted analysis to account for the variance caused by different factors. Statistical heterogeneity is commonly reported in meta-analyses of prevalence data^60,61^. Secondly, only one study had a low risk of bias while 15 of the studies had a medium risk of bias and 3 had a high risk. Thirdly, our results are based on data from only three provinces, lacking data from two other provinces, limiting generalization to the entire country. Finally, our study only included peer-reviewed studies and excluded grey literature, which could have resulted in publication bias.

Despite the limitations, this meta-analysis represents the first study of its kind to find a pooled prevalence of diabetic peripheral neuropathy in Pakistan. Our transparent approach involved publishing a study protocol and utilizing scientific and statistical methods, including subgroup and meta-regression analyses, to account for potential influencing variables in our estimate.

## Conclusion

The estimated prevalence of peripheral neuropathy in patients with diabetes in Pakistan is around 43%. The condition is more common in those who have poorly controlled diabetes and is a major of morbidity and mortality in the Pakistani population. Early detection and treatment of peripheral neuropathy are critical for preventing the condition's progression and complications.

## Supplementary Information

Supplementary Information.

## Data Availability

All data are included in this manuscript and presented in Table 1.
